# Investigating G-quadruplex structures in *RPGR* gene: Implications for understanding X-linked retinal degeneration

**DOI:** 10.1016/j.heliyon.2024.e29828

**Published:** 2024-04-18

**Authors:** Luigi Donato, Concetta Scimone, Simona Alibrandi, Domenico Mordà, Ivan Anchesi, Sergio Zaccaria Scalinci, Carmela Rinaldi, Rosalia D'Angelo, Antonina Sidoti

**Affiliations:** a Department of Biomedical and Dental Sciences and Morphofunctional Imaging, Division of Medical Biotechnologies and Preventive Medicine, University of Messina, Messina, 98125, Italy; b Department of Biomolecular Strategies, Genetics and Cutting-Edge Therapies, I.E.ME.S.T., Palermo, 90139, Italy; c Department of Veterinary Sciences, University of Messina, 98122, Messina, Italy; d IRCCS Centro Neurolesi "Bonino-Pulejo", Via Provinciale Palermo, Contrada Casazza, 98124, Messina, Italy; e Department of Medical and Surgical Sciences, University of Bologna, Bologna, 40121, Italy

**Keywords:** G-quadruplex, RPGR, WGA, Pyridostatin, DNA-Polymerase, RNA-Polymerase

## Abstract

**Aims:**

This pilot study investigates the potential pathogenic role of G-quadruplex (G4) structures in *RPGR*-associated retinal degeneration, starting from a case of suspected X-linked form affected family. We hypothesize that the stabilization of these structures might alter DNA replication and transcription, inducing genetic instability and influencing gene expression.

**Main methods:**

We conducted whole genome amplification experiments and next-generation sequencing to detect the blockade of polymerase activity by G4 structures. Our specific focus was the *RPGR* gene, which hosts a high concentration of predicted G4-forming motifs and is implicated in most X-linked retinal degeneration cases. To understand the potential interference of G4 structures, we applied computational and 3D molecular modeling to visualize interferences in DNA replication and transcription regulation.

**Key findings:**

Our data confirmed the obstruction of DNA polymerase enzymes by G4 structures, particularly when stabilized by the compound pyridostatin. This obstruction was evident in the reduced amplification of *RPGR* gene regions and a shift in the start/end sites of putative G4 motifs. Moreover, the modeling indicated a potential disruption of critical promoter elements and RNA polymerase binding, which could drastically alter gene expression.

**Significance:**

Our findings suggest that G4 formation in the *RPGR* gene could lead to genetic instability and affect the expression of RPGR, contributing to retinal dystrophy. Moreover, this study underscores the broader implications of G4 structures in other genetic disorders. Improved understanding of G4 structures could reveal novel therapeutic targets to combat genetic disorders, promoting the advancement of personalized medicine and precision health.

## Highlights

• WGA and next-gen sequencing revealed G4 clusters obstructing DNA polymerase in RPGR gene regions.
• Computational analyses predicted shifting G4 start/end sites in RPGR between treated and untreated DNA.
• 3D modeling showed stabilized G4 structures hindering DNA/RNA polymerase processes on RPGR.
• Analyses suggest pervasive G4 formation in RPGR causes genetic instability and disrupted expression.
• Integrated approach provides new evidence for involvement of G4 structures in RPGR-linked disorders.

## 1 Introduction

Retinal disorders of a hereditary nature (IRDs) form an extensive and heterogeneous array of genetic ailments that target the retina, thereby becoming a crucial factor in visual impairment scenarios [[1], [2], [3]]. Within this expansive category, Retinitis Pigmentosa (RP) distinguishes itself as the most genetically and clinically multifaceted subgroup, with its occurrence rate being roughly 1 in 4000 in the overall population [4]. Intriguingly, about 15 % of RP instances, specifically the X-linked variants (OMIM 26800), display a more aggressive clinical profile. These instances commonly initiate with early-onset symptoms like difficulty in night vision and a gradual narrowing of the visual field, often advancing to complete vision loss by the third or fourth decade of life [5]. Currently identified as the primary causative genes for X-linked RP are *RP2* (OMIM 312600; Xp11.3) and *RPGR* (OMIM 312619; Xp21.1) [6]. The *RP2* gene is responsible for encoding a GTPase-activating protein (GAP) that consists of 350 amino acids. This protein collaborates with tubulin-specific chaperones to mediate specialized trafficking between the Golgi complex and the ciliary membrane. Variants affecting the protein's N-terminus disrupt its intended cellular targeting [7]. In contrast, *RPGR* encodes a protein featuring six RCC1-like domains (RLDs), which are indicative of evolutionarily conserved guanine nucleotide exchange factors. This protein is predominantly involved in ciliogenesis and has implications in a variety of cellular functions, including but not limited to, photoreceptor integrity, microtubule organization, and primary cilia transport regulation [8]. The genomic layout of *RPGR* is intricate, extending over 150 kb and including 19 exons. This complexity results in alternative splicing events that produce multiple transcript variants, each encoding distinct protein isoforms [9]. The most frequently observed retinal isoform is composed of exons 1–19. A variety of splice variants have been identified across different human tissues, and mutations in the ORF15 exon have been pinpointed in a dog model exhibiting a unique form of X-linked progressive retinal degeneration [10]. The ORF15 exon is especially noteworthy for its repetitive sequences, particularly those abundant in guanine (G), which pose significant challenges for genetic screening [11]. In the current study, we probe into the potential molecular underpinnings that contribute to these screening challenges. We evaluate the possibility that the repetitive nature of the ORF15 exon could give rise to a complex G-Quadruplex (G4) structure. A G4 is a secondary DNA structure comprising two or more stacked G-quartets, which are then stabilized by a monovalent cation [12]. In the genomic context, G4s have been previously identified in numerous regulatory regions, including promoters, CpG islands, 5′UTRs, first exons, first exon/intron junctions, and nuclease-hypersensitive sites, thereby influencing gene expression [13]. Interestingly, sequences capable of forming G4s have been reported to play roles in DNA replication, recombination, and splicing processes [14]. Currently, the identification of G4 structures is primarily conducted through computational analyses, with the main experimental techniques being microarray and ChIP-Seq [15]. Only in the last years a new approach based on Whole Genome Amplification (WGA) and Sequencing (WGS) was exploited [16]. In this investigation, we assess how G4 structures could potentially form on the *RPGR* gene, particularly focusing on its ORF15 exon. We initiate our exploration with the latest bioinformatic tools and then proceed to combine the utility of a DNA polymerase stop assay, mediated by pyridostatin, with the capabilities of WGA and WGS. Pyridostatin Trifluoroacetate Salt (RR82) is recognized for its ability to stabilize G-quadruplex structures with a Kd of 490 nM in multiple cell-free assays [17]. Finally, we conduct a comparative downstream analysis to ascertain whether the presence of variants within *RPGR* could influence the formation of G4 structures.

## 2 Materials and methods

### 2.1 Family clinical data

**Fig. 1:**
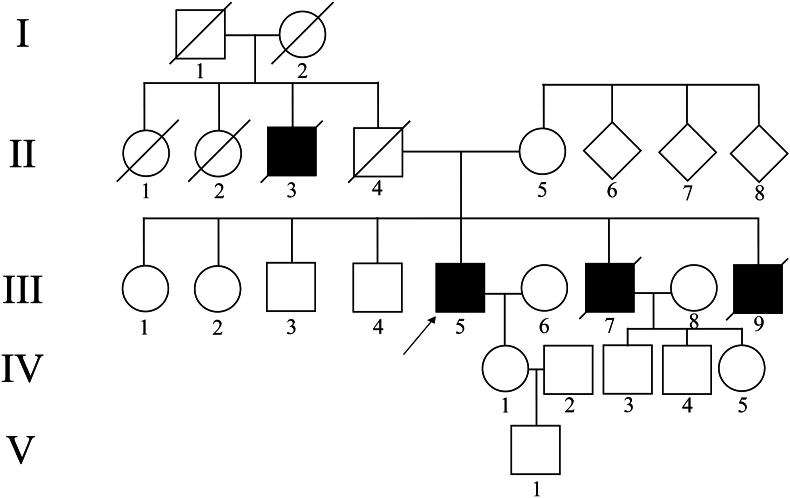
**Pedigree of the Sicilian family affected by a suspected X-linked form of inherited retinal dystrophy**. The affected (black fill) and un-affected (no fill) members are shown. Arrow: proband; circle: female; square: male.

We conducted an examination on a Sicilian family from Palermo, consisting of four members with a suspected X-linked inherited retinal dystrophy, of which only one is still alive (Fig. 1). In addition to ocular problems, the proband reported deafness and asthma. The performed ophthalmological evaluations included tests for dark adaptation, color vision, and visual acuity using the Snellen chart, along with computerized visual field measurements. Furthermore, the proband (III5) underwent a series of advanced diagnostic procedures, including optical coherence tomography (OCT). We utilized the RS-3000 Advance system (Nidek, Aichi, Japan) to perform 3D Spectral-Domain Optical Coherence Tomography (SD-OCT) imaging. This OCT device boasts a depth resolution of 7 μm in tissue and a transverse resolution of 20 μm. Each A-scan reached a depth of 2.1 mm and contained 512 pixels, resulting in a digital depth sampling rate of 4.1 μm per pixel. To achieve wide-area 3D imaging of the posterior pole, we conducted raster scanning over a 6 × 6 millimeter square area, centered on the foveal center. This scanning process involved a density of 512 A-scans horizontally and 128 B-scans vertically. All the OCT images generated provided data on retinal thickness and offered tomographic mapping. We gathered the clinical history of the four affected individuals, paying particular attention to several key aspects. These included their perceived level of vision loss, the age at which symptoms first appeared, the progression of their condition over time, any medications they were taking, and other clinical manifestations specific to inherited retinal diseases. These specific manifestations included impairment of night vision, loss of peripheral vision, and changes in color perception. Blood samples were, finally, collected from the proband and other healthy family members (II5, III1, III2, III3, III4) for genomic DNA sequence analysis. The research followed the tenets of the Declaration of Helsinki and was approved by the Scientific Ethics Committee of the Azienda Ospedaliera Universitaria *–* Policlinico “G. Martino” Messina (protocol number 0014661). Informed consent was signed by all family members and controls after explanation of the nature and possible consequences of the study.

### 2.2 X-linked IRD known genes genotyping

To ascertain the potential presence of X-linked IRD within the family under study, we conducted genotyping analyses focusing on *RP2* and *RPGR* genes, which are already established as causative agents for X-linked forms of the disorder. Genomic DNA was isolated from the peripheral blood samples of family members utilizing a QIAamp DNA Blood Midi Kit (Qiagen, Hilden, Germany), in compliance with the guidelines provided by the manufacturer. Primer sequences for PCR are available upon request. The PCR reactions were executed in 50 μl reaction mixtures, comprising 0.2 μm of each primer, 1.5 U of MyTaq DNA Polymerase (Bioline, London, UK), and 0.8 μg of genomic DNA serving as the template. The PCR conditions were set as follows: an initial denaturation at 95 °C for 1 min, followed by 35 cycles of 15-s denaturation at 95 °C, 15-s annealing, and 10-s extension at 72 °C. Annealing temperatures were individually optimized for each set of primers. Sanger sequencing was employed for molecular analysis, and the sequencing was performed on a 3500 Genetic Analyzer (Applied Biosystems, ThermoFisher Scientific, Monza, Italy) using BigDye Terminator v3.1 chemistry, as per manufacturer's instructions.

### 2.3 Inhibitory activity test of pyridostatin on PCR

To investigate the potential influence of G-Quadruplex structures in the unidentified X-linked form within the family, we initiated an experimental procedure. The aim was to stabilize these structures to better understand their possible role in IRD. For this purpose, we selected pyridostatin, a compound known for its reliable G4-stabilizing properties, as previously demonstrated in other studies [18]. To gauge the effectiveness of pyridostatin, we considered two distinct groups of genomic regions: the first group included G4-forming regions such as c-KIT, c-MYC, and VEGFA, while the second group consisted of non-G4-forming regions like SOD1, CD4, and CNDP2. The sequences of the primers used are available upon request. PCR reactions were set up in 50 μl volumes containing either 0.1 μM, 1 μM, or 5000 μM (stock solution) of Pyridostatin (RR82) Trifluoroacetate Salt (Selleckchem), along with 2 μl of each primer (10 μM), 0.8 μg of human genomic DNA, 1.5 U of MyTaq DNA Polymerase (Bioline, London, UK), and its accompanying buffer (10 μl). Control reactions were also executed, which contained the same concentrations of all reagents but excluded pyridostatin. Post-PCR, 5 μl of the amplified material was subjected to electrophoresis on a 2 % agarose gel for analysis.

### 2.4 Whole genome amplification of pyridostatin-treated and untreated sample

WGA was performed on two proband's DNA samples, one treated and one untreated with pyridostatin, using the REPLI-g Mini Kit (Qiagen) as per the manufacturer's protocol. Briefly, 100 ng of DNA from each sample was mixed with denaturation buffer and incubated at room temperature for 3 min. This was followed by the addition of neutralization solution and a master mix containing phi29 DNA polymerase, with or without pyridostatin (1 μM). The samples were then incubated at 30 °C for 16 h, followed by enzyme inactivation at 65 °C for 3 min. Control DNA from a healthy family component (III6) was also amplified under similar conditions, and each experiment was replicated thrice. The WGA products thus obtained were subsequently used as templates for Whole Genome Sequencing (WGS).

### 2.5 Whole Genome Sequencing of the WGA products

For sequencing, libraries were constructed utilizing the TruSeq Nano DNA Kit (Illumina) and the sequencing was executed on the NovaSeq 6000 platform (Illumina) in a paired-end mode, with each read being 150 bp in length, achieving a total sequencing coverage of 110 Gb. The raw data generated were then subjected to quality assessment using the FastQC tool (version 0.11.9) and reads with a Phred score less than 30 were trimmed, along with the removal of adaptor sequences. The sequencing data were subsequently processed in-house using the CLC Genomics Workbench 23.0.1 software, as has been our practice in other recently published works [19,20]. The analytical workflow included alignment to the GRCh38 Human Reference Genome, allowing for fewer than 3 mismatches per 100 bp in each alignment. This was followed by the removal of duplicate reads, realignment of InDels, and Base Recalibration before proceeding to variant calling. The Fixed Ploidy Variant Caller was employed for variant calling, which reports variants with a probability greater than 90 %. This caller combines a Bayesian model to examine posterior probabilities with a maximum likelihood methodology. Finally, the identified variants were annotated using the ANNOVAR tool version 20221231 and included in databases. Visualization of the mapped reads and the predicted locations of G4 quadruplex sites was carried out using the Integrative Genome Viewer and the USCS Genome Browser.

### 2.6 In silico explorative analyses of RPGR putative G4-quartets

We used a variety of prediction software methods to investigate the potential consequences of different mutations in the human *RPGR* gene that might result in the stability of G-quadruplex DNA structures. For the precise identification and study of G-quadruplex structures, each of these tools provides particular and specialized algorithms. pqsfinder is an algorithm created by Hon et al. that is specifically made to locate genomic PQS [21]. It makes use of the sliding window method and afterwards assesses overlapping oligonucleotide substrates, enabling predictions of more complicated and heterogeneous G-quadruplexes. A web-based program called QGRS Mapper maps quadruplexes that create G-rich sequences (QGRS) in nucleotide sequences [22]. It offers a significant chance to examine both coding and noncoding sequences to find possible G-quadruplex motifs. G4P Calculator is a special tool that forecasts the likelihood that G-quadruplexes will form within DNA sequences using empirical and thermodynamical data [23]. For shedding light on the potential thermodynamic stability of these structures, it is very beneficial. The G4 Hunter method employs a variable window size approach to assess and identify the potential for G-quadruplex formation. The G4 Hunter grading system is based on a weight function that takes both G-richness and G-skewness into account [24]. G4 Catchall is a program that explicitly accounts for the G-quadruplexes' variable loop lengths and provides a broader method of identifying probable quadruplex sequences by analyzing G-tracts and supplementary loops [25]. A unique tool for predicting intramolecular G-quadruplex (imGQ) structures in nucleotide sequences is imGQfinder. It offers a thorough examination of these structures, including their folding and thermodynamic stability [26]. To find probable nucleic acid structures, such as G-quadruplexes, this software, 3D Nus, use 3D structure prediction methods. It helps to comprehend the potential spatial orientation of these motifs as well as their 3D structure [27]. Finally, the assessment of potential docking between RPGR DNA gene tract and DNA or RNA polymerases, as well as the dynamic molecular modeling of their complex, was conducted using the integrated molecular design platform, SAMSON-Connect (https://www.samson-connect.net). This platform leverages various extensions including AutoDock Vina Extended [28], FIRE state updater [29], GROMACS Model Generator [30] and Ligand Path Finder [31] to provide comprehensive insights. Before engaging these extensions, each structural model was carefully validated. This initial validation process involved identifying any small free molecules and clashes, such as superimposed and covalently docked bound ligands with each other or the protein. Additionally, the process involved checking the bond length and removing any alternate locations, ensuring the model's suitability for the subsequent steps.

## 3 Results

### 3.1 The clinical examination of family proband highlighted a typical ocular phenotype of X-linked IRD

**Fig. 2:**
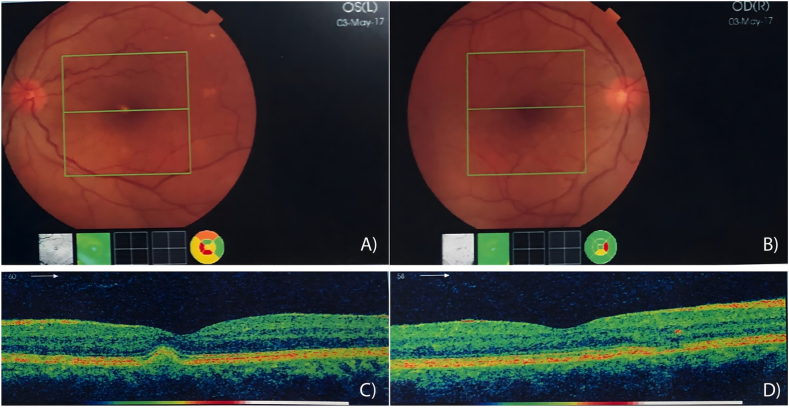
**Proband's ophthalmological examination**. The fundus (A, B) and OCT (C, D) analyses of proband's eyes highlighted a typical pattern of X-linked IRD phenotype, with retinal thinning, alteration of ellipsoid zone and a peripapillary atrophy.

Ophthalmological features of the family proband were typical of those associated to X-linked IRDs, such as night blindness from birth, and progressive loss of visual field and visual acuity, as well as myopia. Fundus examination showed smaller and tilted optic disc with peripapillary atrophy. The OCT scan through the retina of the proband showed reduced contour of the foveal surface and diminished outer nuclear layer, accompanied by the compromission of the ellipsoid zone (Fig. 2). The other family members, instead, showed no ocular symptoms and evidenced no alteration at the instrumental examination.

### 3.2 RP2 and RPGR genotyping evidenced no significant variants known to be associated or causative of X-linked IRDs

The Sanger sequencing of both *RP2* and *RPGR* genes highlighted only two intronic variants in the first gene (rs1556327486 and rs200266931), and a list of several ones, mostly intronic, carried by *RPGR* (see following sub-chapters for the details). Electropherograms are available upon request. Thus, we proceeded with the G4 hypothesis.

### 3.3 Investigating the inhibitory activity of a G4 ligand on DNA polymerase extension in G4-Forming sequences

**Fig. 3:**
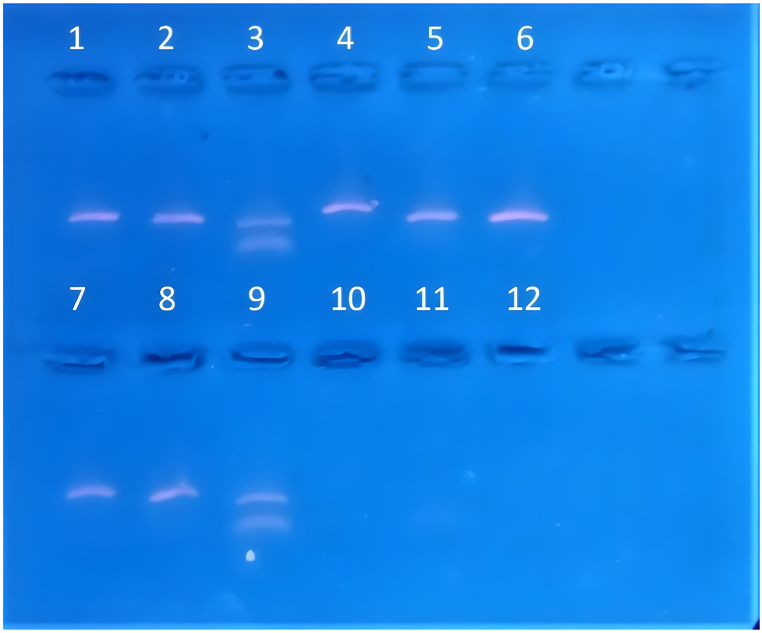
**Pyridostatin G4 inhibitory activity test.** The activity of pyridostatin was evaluated on non-G4-forming region genes (*CNDP2*, *CD4 and SOD1*) and on G4 known forming region (*c-KIT, c-MYC* and *VEGFA*). *CNDP2* (lane 1); *CD4* (lane 2); *SOD1* (lane 3); *C-KIT* (lane 4); *C-MYC* (lane 5); *VEGFA* (lane 6); *CNDP2* con RR82 (lane 7); *CD4* treated with RR82 (lane 8); *SOD2* treated with RR82 (lane 9); *C-KIT* treated with RR82 (lane 10); *C-MYC* treated with RR82 (lane 11); *VEGFA* treated with RR82 (lane 12).

The first aim of this work was to confirm the inhibitory activity of the G4 ligand, specifically the pyridostatin (RR82), against DNA polymerase extension on G4-forming sequences. RR82 binds to the top of the G-tetrad structure through π-stacking and electrostatic interactions. To assess its effect on DNA polymerase extension, polymerase chain reaction (PCR) experiments were conducted using human genomic DNA in the presence of RR82. PCR primers targeting G4-forming regions (*VEGFA, c-MYC* and *c-KIT*) and non-G4-forming regions (*SOD1, CD4* and *CNDP2*) in human genomic DNA were designed. Without the presence of RR82, PCR successfully amplified all targeted regions. However, when amplifying G4-forming regions in the presence of 1 μM RR82, no amplification was observed, while amplification of non-G4-forming regions still occurred (Fig. 3). This indicates that pyridostatin specifically inhibits DNA polymerase extension on G4-forming regions during PCR. Thus, the produced results confirmed the inhibitory effect of RR82, a G4 ligand, on DNA polymerase extension specifically on G4-forming regions during PCR amplification.

### 3.4 Effect of RR82 on whole genome amplification: analysis of DNA polymerase extension and product yield

**Fig. 4:**
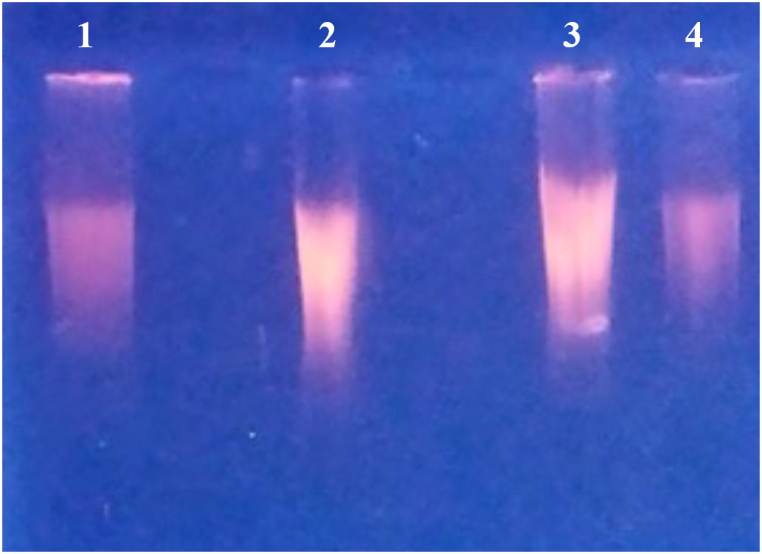
**Effect of RR82 on Whole Genome Amplification**. The pyridostatin treatment highlight important difference in WGA. WGA of proband's DNA treated RR82 (line 1); WGA of proband's DNA (line 2); WGA of control DNA (line 3); WGA of control DNA treated with RR82 (line 4).

WGA was executed with the inclusion of the G4 ligand RR82, utilizing the multiple displacement amplification technique mediated by Phi29 DNA polymerase. This method typically yields amplified products exceeding 10 kb in length. In the absence of RR82, the WGA process resulted in products with an approximate length of 23 kb, as illustrated in Fig. 4. Conversely, when 1 μM of RR82 was incorporated into the WGA reaction, no amplified products were discernible upon 1 % agarose gel electrophoresis. Subsequent G4-seq analysis identified as many as 716,310 G4-forming sequences within the human genome that are stabilized by the G4 ligand. These observations imply that the activity of Phi29 DNA polymerase could be hindered by the multitude of G4-forming regions present in genomic DNA, leading to a reduced yield of WGA products.

### 3.5 Identification of G4 clusters in human genomic DNA through high-throughput sequencing of whole genome amplification products

To pinpoint the locations of G4-forming sequences within the human genome, we conducted high-throughput sequencing of the WGA products using the Illumina HiSeq X10 platform in paired-end mode (150 bp x2). The WGA products served as the direct templates for this sequencing, bypassing any purification steps. This was because the presence of RR82 would inhibit DNA polymerase-based reactions on G4-forming regions during both library preparation and sequencing. The sequencing generated 337 million reads (equivalent to a 35× depth of coverage) for the RR82 library and 311 million reads (corresponding to a 32× depth of coverage) for the control library. Initially, the number of mapped reads was calculated for each 200 bp window across the entire genome, with a sliding window size of 200 bp. In line with our PCR analysis of WGA products, we observed a reduction in the number of mapped reads in the RR82 library specifically in the *c-MYC*, *c-KIT*, and *VEGFA* G4 regions (as shown in Table 1). However, no specific peaks were observed at the G4-forming sequences, as the reduction in mapped reads was observed across the entire region. Our sequencing data further indicated that the average depth of coverage for the RR82 library was less than that for the control library. Subsequently, we counted the mapped reads in 10 kbp windows with a 10 kbp sliding window size, based on the highest potential quadruplex sequence (PQS) frequencies and the ratios of reads in the RR82 library compared to the control library. These observations suggest that analyzing the mapped reads over large genomic windows could facilitate the identification of clusters of G4-forming sequences, likely induced by the presence of a G4 ligand. Specifically, in the proband's untreated sample, we identified 24 variants (22 intronic and 2 coding) carried by *RPGR*, whereas in the pyridostatin-treated sample, we found only 7 intronic variants within the same gene, all of which were distinct from those in the untreated sample (as detailed in Table 2). This result should reflect the consequence of the pyridostatin treatment, determining the loss of G4-forming regions amplification and, then, of the related sequence data. The control sample show no variants in both conditions, even if the full sequence was normally obtained in both cases, suggesting a potential role in the proband's retinal compromission.

**Table 1:** **FastQ raw data statistics.** GC (%): GC content. Total Yield (bp): Total number of bases sequenced. Total reads: Total number of reads. Q20 (%): Ratio of bases with a phred quality score greater than 20. Q30 (%): Ratio of bases with a phred quality score greater than 30.

Sample	Total yield	Total reads	GC%	Q20 %	Q30 %
Proband's Untreated WGA	117,650,067,218	779,139,518	46.41	95.96	90.17
Proband's RR82 Treated WGA	93,294,596,812	617,845,012	32.35	93.47	86.38

**Table 2:** **Variants found in *RPGR* gene by WGA/WGS of pyridostatin treated and untreated proband's DNA samples.** The table focused on proband's DNA samples only because, as already stated within the text, the healthy control sample did not show any variant within *RPGR*.

PROBAND’S UNTREATED DNA								
Region	Zygosity	MAF	Strand	Variant Consequence	dbSNP	Exon/Intron Number	Amino acid change in longest transcript	Coding region change in longest transcript
38272607^38272608	Heterozygous	/	+	splice pyrimidine tract	/	Intron 27	/	c.2856 + 793dupT
38273982	Homozygous	0.718	–	intron	rs2301276	Intron 27	/	c.2765-505A > C
38277830	Homozygous	0.536	+	intron	rs5917556	Intron 27	/	c.2521-1058A > G
38284619	Heterozygous	/	+	3′UTR	/	Intron 23	/	c.*921C > A
38285869	Homozygous	/	+	intron	/	Intron 23	/	c.2520 + 1225G > T
38287748	Homozygous	/	+	missense	/	Exon 22	p.Glu622Asp	c.1753 + 113A > C
38293396	Homozygous	0.166	+	intron	rs12834659	Intron 19	/	c.1415–1912T > G
38299037	Homozygous	0.165	–	synonymous	rs1801686	Exon 16	/	c.1164G > A
38302239	Heterozygous	/	+	intron	/	Intron 14	/	c.935-868T > G
38304165	Homozygous	0.526	+	intron	rs7058254	Intron 12	/	c.934 + 470T > C
38308741	Homozygous	0.526	+	intron	rs7064372	Intron 10	/	c.778 + 1874G > T
38309098	Homozygous	0.199	+	intron	rs34403375	Intron 10	/	c.778 + 1517T > C
38309731	Homozygous	0.491	+	intron	rs28431080	Intron 10	/	c.778 + 884C > T
38310060	Homozygous	0.527	+	intron	rs5963396	Intron 10	/	c.778 + 555C > G
38310878	Homozygous	0.201	+	intron	rs35019417	Intron 9	/	c.620-105G > C
38312802	Homozygous	0.504	+	intron	rs5963399	Intron 9	/	c.620–2029T > C
38312989	Homozygous	0.731	+	intron	rs5917559	Intron 9	/	c.620–2216A > G
38313286	Homozygous	0.199	+	intron	rs5963400	Intron 9	/	c.620–2513C > T
38315518	Homozygous	0.731	+	intron	rs5917196	Intron 9	/	c.619 + 1798C > G
38315788	Homozygous	0.388	+	intron	rs12841053	Intron 9	/	c.619 + 1528G > A
38315860^38315861	Heterozygous	/	+	intron	/	Intron 9	/	c.619 + 1474_619 + 1477dupCTCT
38316301^38316302	Homozygous	/	+	intron	rs34282277	Intron 9	/	c.619 + 1016dupC
38316684	Homozygous	/	+	intron	/	Intron 9	/	c.619 + 632G > C
38318766	Homozygous	0.295	+	intron	rs3810692	Intron 7	/	c.469 + 63C > T
**PROBAND’S DNA TREATED WITH PYRIDOSTATIN**								
**Region**	**Zygosity**	**MAF**	**Strand**	**Variant Consequence**	**dbSNP**	**Exon/Intron Number**	**Amino acid change in longest transcript**	**Coding region change in longest transcript**
38291992	Heterozygous		+	intron	/	Intron 19	/	c.1415-508A > T
38294997	Heterozygous	/	+	intron	/	Intron 19	/	c.1414 + 2287A > T
38295524	Heterozygous	/	+	intron	/	Intron 19	/	c.1414 + 1760T > C
38303241	Heterozygous	/	+	intron	/	Intron 13	/	c.934 + 1394T > C
38319342	Homozygous	/	+	intron	/	Intron 6	/	c.311-355G > A
38319534	Homozygous	0.491	+	intron	rs12842301	Intron 6	/	c.311–547C > T
38319937	Heterozygous	/	+	intron	/	Intron 6	/	c.311–950A > T

### 3.6 Correlation between detected changes and predicted PQS

In our analysis, we sought to establish a correlation between the genomic variants detected in the *RPGR* gene (Table 2) and the predicted PQS (Table 3, Table 4, Table 5, Table 6, Table 7). Our statistical analysis focused on comparing the positions of the 24 variants identified within *RPGR* in the untreated sample, which included 22 intronic and 2 coding variants, with the positions of PQS predicted by tools such as pqsfinder, QGRS Mapper, and others (more details within the next paragraph). Notably, in the pyridostatin-treated sample, we observed a reduction to only 7 intronic variants carried by *RPGR*, all distinct from those in the untreated sample. This discrepancy underscores the impact of pyridostatin in stabilizing G4 structures, subsequently influencing genomic amplification and variant detection.

**Table 3:** G4 clusters found by pqsfinder in wildtype control sample, treated and untreated proband's DNA.

pattern	strand	score	nt	nb	nm	rl1	rl2	rl3	ll1	ll2	ll3	WILDTYPE and TREATED		UNTREATED	
												start	end	start	end
[CCC]T[CC(TACACACA)C][CCC]ACTA[CCC]	–	48	3	1	0	3	11	3	1	0	4	12554	12578	12559	12583
[GGGG]ATATAAGTAAATGAGTT[GGGG]TTAT[GGG(AACT)G][GGGG]	+	68	4	1	0	4	4	8	17	4	0	13607	13647	13612	13652
[CCCC]AGACTAGAGGAAAATT[CCCC]T[CC(TTTGAA)CC]ATATTAACA[CCCC]	–	62	4	1	0	4	4	10	16	1	9	25854	25901	25859	25906
[GGG(AGTGCAC)G]AGTGTAGCT[GGGG][GtGG]A[GGGG] **[GGG(AGTGCAC)G]AGTGTAGCT[GGGG][GtGG]T[GGGG] (TREATED)**	+	54	4	1	1	11	4	4	9	0	1	36125	36157	36130	36162
[GGaG][G(A)GGG]A[GGGG]A[GG(A)GG]	+	47	4	2	1	4	5	4	0	1	1	41327	41346	41332	41351
[GGGG]AAGGA[GG(AGAAA)GG]AGAGGAAGAA[GG(AGACCAA)GG]AGA[GGGG]	+	50	4	2	0	4	9	11	5	10	3	41489	41534	41494	41539
[GGGG]AGA[GGGG]A[GG(AAAAAGA)GG]AGGGA[GGGG]	+	83	4	1	0	4	4	11	3	1	5	41552	41583	41557	41588
[GGGG(AAAGGA)G]AGA[GGG(AAGA)GG]AAGA[GG(AGGA)GGG]TGA[GGGGG]	+	80	5	3	0	11	9	9	3	4	3	41609	41652	41614	41657
[G(AA)GGGG]A[GGGGG]AAGA[GG(AGGAAG)GGG]A[GGGGG]	+	108	5	2	0	7	5	11	1	4	1	41661	41694	41666	41699
[GG(AA)GG]A[G(AA)GGG]AAA[GGGG]AGGAAGAA[GGGG]	+	60	4	2	0	6	6	4	1	3	8	41702	41733	41707	41738
[GGGG]AGGAAGAA[GGGG]AGGAAGGAGAA[GGGG]A[GGGG]	+	90	4	0	0	4	4	4	8	11	1	41745	41780	41750	41785
[GGGG]A[GGG(AGAA)G]A[GG(AA)GG]AGAA[GGGG]	+	68	4	2	0	4	8	6	1	1	4	41799	41826	41804	41831
[GGGG]A[GGG(AGAA)G]A[GG(AA)GG]AGAA[GGGG]	+	68	4	2	0	4	8	6	1	1	4	41850	41877	41855	41882
[GG(AA)GG]A[G(AA)GGG]AAA[GGGG]A[GG(A)GG]	+	50	4	3	0	6	6	4	1	3	1	41891	41916	41896	41921
[GGGG]A[GGGG]GAAGAGGAGGAAGGAGAA[GGGG]AA[GGGG]	+	89	4	0	0	4	4	4	1	18	2	41934	41970	41939	41975
[GGGGG]AAGA[GGaGG]AA[GG(AGAAT)GGG]A[GGGGG]	+	98	5	1	1	5	5	10	4	2	1	41987	42018	41992	42023
[GGGG]A[GGGG]GAAGAGGAAGGAGAA[GGGG]AA[GGGG]	+	92	4	0	0	4	4	4	1	15	2	42036	42069	42041	42074
[GGGGG]AAGA[GGaGG]AA[GG(AGAAG)GGG]A[GGGGG]	+	98	5	1	1	5	5	10	4	2	1	42086	42117	42091	42122
[GGGG]AAGAAGAA[GGGG]A[GG(AAGAA)GG]A[G(A)GGG]	+	62	4	2	0	4	4	9	8	1	1	42129	42160	42134	42165
[GGGG]A[GGG(A)G]AA[GGGG]A[GG(AAGAAGA)GG]	+	71	4	2	0	4	5	4	1	2	1	42171	42198	42176	42203
[GGG(AAGT)GG]AA[GGGG(AGGTG)G]AA[GGGG(AGGAAG)G]AGA[GGGGG]	+	84	5	3	0	9	10	11	2	2	3	42202	42243	42207	42248
[GGG]TTA[GGG]GT[GGG]TT[GGG]	+	67	3	0	0	3	3	3	3	2	2	44984	45002	44989	45007
[CCCC]TCT[CCCC]AGTT[CCC(ATGGCT)C][C(TT)CCC]	–	66	4	2	0	4	4	10	3	4	0	47689	47719	47694	47724
[CCCaC]T[C(TCAG)CCCC]AC[CCCCC]AG[CCCCC]	–	101	5	1	1	5	9	5	1	2	2	48577	48605	48582	48610
[CtCC]ATT[CCCC]TT[CCCC]TTTTT[CCC(TAA)C]	–	54	4	1	1	4	4	4	3	2	5	52382	52410	52387	52415

**Table 4:** **G4 clusters found by QGRS Mapper in wildtype control sample, treated and untreated proband's DNA.** The grey background and bold characters highlight the differences between Wildtype/Treated and Untreated samples.

Position	Length	QGRS (WILDTYPE and TREATED)	G-Score	Position	Length	QGRS (UNTREATED)	G-Score
875	24	GGATCGGCCCTGTAGGGGGACAGG	17	892	19	GGACAGGCCCCAGGCTTGG	19
904	19	GGCTTGGCAGCCGGACTGG	19	916	21	GGACTGGGGCGGGATCGCTGG	18
960	18	GGCAGAGGACCGGACGGG	20	960	18	GGCAGAGGACCGGACGGG	20
**985**	**25**	**GGATGGGGGAGAACGGGAAAGAGGG**	**20**	**985**	**24**	**GGATGGGGGAGAACGGGAAAGAGG**	**20**
**6382**	**17**	**GGCAGGTTAGGTAATGG**	**19**	**6376**	**17**	**GGTGATGGCAGGTTAGG**	**19**
**6666**	**30**	**GGTTTCACCATGTTGCCCAGGTTGGTCTGG**	**6**	**6397**	**20**	**GGATCAGATTGGTGAGGAGG**	**14**
**/**	**/**	**/**		**6666**	**30**	**GGTTTCACCATGTTGCCCAGGTTGGTCTGG**	**6**
**7414**	**24**	**GGTGAGTGGAAGGCACCCAGAAGG**	**14**	**7413**	**25**	**GGGTGAGTGGAAGGCACCCAGAAGG**	**14**
12371	28	GGCAATAATCACATCATGGAGAATGGGG	6	12376	28	GGCAATAATCACATCATGGAGAATGGGG	6
12989	25	GGTTTCACCATGTTGGTCAGGCTGG	11	12994	25	GGTTTCACCATGTTGGTCAGGCTGG	11
13629	23	GGGTTATGGGAACTGGGGGCAGG	21	13634	23	GGGTTATGGGAACTGGGGGCAGG	21
13719	25	GGGGAAGGTAGCTGTCAGAGCCAGG	6	13724	25	GGGGAAGGTAGCTGTCAGAGCCAGG	6
14410	15	GGAGAGAGGTGGTGG	17	14415	15	GGAGAGAGGTGGTGG	17
14581	17	GGAAGAGGAGGATGTGG	18	14586	17	GGAAGAGGAGGATGTGG	18
14923	29	GGGGCGTCAGGACATATTTCAGAGAAAGG	5	14928	29	GGGGCGTCAGGACATATTTCAGAGAAAGG	5
**15126**	**25**	**GGTTAGGGATCATGGATCCTAAAGG**	**17**	**15129**	**27**	**GGGGTTAGGGATCATGGATCCTAAAGG**	**18**
15229	27	GGCTGGAGTGCAGTGGCATAATCACGG	14	15234	27	GGCTGGAGTGCAGTGGCATAATCACGG	14
15556	29	GGTTGGGGAGTAGATAGAAATATGACTGG	2	15561	29	GGTTGGGGAGTAGATAGAAATATGACTGG	2
15863	27	GGCAACAAGCATGGGAAAATGGAAGGG	14	15868	27	GGCAACAAGCATGGGAAAATGGAAGGG	14
16333	27	GGGGATTTAGGGGGCTGGTCATGTAGG	19	16338	27	GGGGATTTAGGGGGCTGGTCATGTAGG	19
16553	14	GGGGTTGGGCAAGG	18	16558	14	GGGGTTGGGCAAGG	18
16851	28	GGAACTCCTTGGTAAGAGTCTTTGGAGG	11	16856	28	GGAACTCCTTGGTAAGAGTCTTTGGAGG	11
17032	20	GGTTTTGGGGATACCATAGG	13	17037	20	GGTTTTGGGGATACCATAGG	13
17503	28	GGTGGAGAGCATACTGTGGTTCTCACGG	10	17508	28	GGTGGAGAGCATACTGTGGTTCTCACGG	10
17937	29	GGGAGGCCAGGAGATCTCTTGAGCCCAGG	7	17942	29	GGGAGGCCAGGAGATCTCTTGAGCCCAGG	7
**18027**	**26**	**GGTCTCAGGTATTCAGGAGGCTGAGG**	**19**	**18039**	**26**	**GGTATTCAGGAGGCTGAGGTGGGAGG**	**19**
**18073**	**19**	**GGGGAGGTTGAGCTTGAGG**	**11**	**18078**	**19**	**GGGGAGGTTGAGGTTGAGG**	**20**
18491	25	GGTTTCGCCATGTTGGCAAGGCTGG	11	18496	25	GGTTTCGCCATGTTGGCAAGGCTGG	11
20020	25	GGGGGAATACAGGAGAAAAACTTGG	12	20025	25	GGGGGAATACAGGAGAAAAACTTGG	12
20406	20	GGTGAGGAACCTGGTTCTGG	19	20411	20	GGTGAGGAACCTGGTTCTGG	19
20836	29	GGGATCAGGGAAACCATGGACCACTGTGG	19	20841	29	GGGATCAGGGAAACCATGGACCACTGTGG	19
20876	29	GGCTGGAACTTGGGACACACTAAGTGAGG	10	20881	29	GGCTGGAACTTGGGACACACTAAGTGAGG	10
20977	30	GGGGTTCACAATGAAGTCTGGGTCCTGAGG	6	20982	30	GGGGTTCACAATGAAGTCTGGGTCCTGAGG	6
22426	27	GGCTGGAGTGCAGTGGCGTGATCTTGG	14	22431	27	GGCTGGAGTGCAGTGGCGTGATCTTGG	14
22757	27	GGCTGGAGTGCAGTGGTGCAATCTTGG	14	22762	27	GGCTGGAGTGCAGTGGTGCAATCTTGG	14
22903	23	GGGGTTTCACCATGTTGGCCAGG	9	22908	23	GGGGTTTCACCATGTTGGCCAGG	9
23381	26	GGACAATTTGGTCAGCTGGGTCTTGG	19	23386	26	GGACAATTTGGTCAGCTGGGTCTTGG	19
24376	23	GGTATCTGGCTAGGGAAACTAGG	19	24381	23	GGTATCTGGCTAGGGAAACTAGG	19
24794	18	GGAGGGCAAGTGGTGTGG	18	24799	18	GGAGGGCAAGTGGTGTGG	18
25044	25	GGAAGAAGGGATAAAGGTGATAAGG	20	25049	25	GGAAGAAGGGATAAAGGTGATAAGG	20
25211	15	GGAGGCTGAGGCAGG	18	25216	15	GGAGGCTGAGGCAGG	18
25939	29	GGAGCGGCTTGAAGAAAGGAGTGTGAAGG	14	25944	29	GGAGCGGCTTGAAGAAAGGAGTGTGAAGG	14
26059	21	GGACCCAGGTTTTGGTATAGG	20	26064	21	GGACCCAGGTTTTGGTATAGG	20
**26269**	**15**	**GGGTGGAGGCCAAGG**	**18**	**26275**	**14**	**GGTGGAGGCCAAGG**	**18**
26403	29	GGAAAATAGACTGTGTTGGGATGTAGGGG	6	26408	29	GGAAAATAGACTGTGTTGGGATGTAGGGG	6
26797	26	GGAGATGGTCGCCACGGAAAATTAGG	18	26802	26	GGAGATGGTCGCCACGGAAAATTAGG	18
27311	15	GGCAGGGCACGGTGG	19	27316	15	GGCAGGGCACGGTGG	19
27351	16	GGGAGGCCAAGGCGGG	19	27356	16	GGGAGGCCAAGGCGGG	19
27522	24	GGTGGAGGTTGCAGTGAGCTGAGG	8	27527	24	GGTGGAGGTTGCAGTGAGCTGAGG	8
27896	15	GGTCGGGCGCGGTGG	19	27901	15	GGTCGGGCGCGGTGG	19
27936	30	GGGAGGCCGAGGCGTGCAGATCAACTGAGG	7	27941	30	GGGAGGCCGAGGCGTGCAGATCAACTGAGG	7
28036	14	GGCGTGGTGGCAGG	19	28041	14	GGCGTGGTGGCAGG	19
29003	26	GGTTGCTTGTGGTGGATGTCACATGG	13	29008	26	GGTTGCTTGTGGTGGATGTCACATGG	13
29902	27	GGCTGGGGTGCAGTGGCGCAATCTTGG	16	29907	27	GGCTGGGGTGCAGTGGCGCAATCTTGG	16
30051	25	GGTTTCACCATGCTGGCCAGGCTGG	11	30056	25	GGTTTCACCATGCTGGCCAGGCTGG	11
31331	22	GGACTATGGAGTTAAACTGGGG	12	31336	22	GGACTATGGAGTTAAACTGGGG	12
32242	27	GGGGTAGGACAAGAAACCAGGACCAGG	14	32247	27	GGGGTAGGACAAGAAACCAGGACCAGG	14
32373	23	GGAAGCTAGGCAAAGGGTTGAGG	19	32378	23	GGAAGCTAGGCAAAGGGTTGAGG	19
32409	30	GGAACTCAGGTAAGAATTGTGGGCCATGGG	17	32414	30	GGAACTCAGGTAAGAATTGTGGGCCATGGG	17
32822	18	GGAGGAAAGTGGATCAGG	17	32827	18	GGAGGAAAGTGGATCAGG	17
**32968**	**28**	**GGACAACCAGTGGGGCTAGAAAGTGAGG**	**10**	**32948**	**27**	**GGTTTGGAGGCAGTTAAGAAGAAAAGG**	**7**
				**32984**	**23**	**GGGGCTAGAAAGTGAGGGAAAGG**	**10**
33265	27	GGACCAAGGTGTTTGCAGTGGAGATGG	15	33270	27	GGACCAAGGTGTTTGCAGTGGAGATGG	15
33673	15	GGCTGAGGTAGGAGG	18	33678	15	GGCTGAGGTAGGAGG	18
33913	12	GGCAGGAGGTGG	20	33918	12	GGCAGGAGGTGG	20
34004	26	GGATTTGGTATAATGGAGTTTACTGG	17	34009	26	GGATTTGGTATAATGGAGTTTACTGG	17
34101	22	GGGGAATGAGGGATTGAAGAGG	13	34106	22	GGGGAATGAGGGATTGAAGAGG	13
**34181**	**26**	**GGTCTGGCAGCTAGAGGGGGATGTGG**	**16**	**34186**	**27**	**GGTCTGGCAGCTAGAGGGGGATGTGGG**	**16**
34321	11	GGAGGGGCCGG	19	34326	11	GGAGGGGCCGG	19
				**34731**	**24**	**GGTAATAGGTAGTATTATGGTAGG**	**14**
35610	27	GGATTTAAAGGGGAGGGATCAGTGTGG	17	35615	27	GGATTTAAAGGGGAGGGATCAGTGTGG	17
35931	18	GGGTGGTTCTGGCCCAGG	19	35936	18	GGGTGGTTCTGGCCCAGG	19
36045	16	GGCCATTGGCAGGAGG	17	36050	16	GGCCATTGGCAGGAGG	17
**36148**	**11**	**GGTGGAGGGGG**	**21**	**36150**	**11**	**GGGGGTGGAGG**	**21**
36276	27	GGACCAACCTGGTATAATGGGCAAGGG	18	36281	27	GGACCAACCTGGTATAATGGGCAAGGG	18
36327	21	GGACGTTGTAGGTCATTGGGG	13	36332	21	GGACGTTGTAGGTCATTGGGG	13
37872	29	GGGATTAGAGGATTAGGAAAGTAAATTGG	15	37877	29	GGGATTAGAGGATTAGGAAAGTAAATTGG	15
39064	15	GGCCAGGCATGGTGG	19	39069	15	GGCCAGGCATGGTGG	19
39104	30	GGGCGGCCAAGATGGGCAGATCACTTGAGG	11	39109	30	GGGCGGCCAAGATGGGCAGATCACTTGAGG	11
39253	30	GGAGAATCGCTGGAACCCAGGAGGCAGAGG	18	39258	30	GGAGAATCGCTGGAACCCAGGAGGCAGAGG	18
39467	27	GGCTGGAGTGCAGTGGCACAATCTTGG	14	39472	27	GGCTGGAGTGCAGTGGCACAATCTTGG	14
39613	22	GGTTTTCACCAGGTTGGCCAGG	14	39618	22	GGTTTTCACCAGGTTGGCCAGG	14
40296	19	GGCCAGGTGGGACCTCAGG	17	40301	19	GGCCAGGTGGGACCTCAGG	17
**40394**	**27**	**GGAAATAGAAAAGGAAAGTGATGGAGG**	**12**	**40399**	**27**	**GGACATAGAAAAGGAAAGTGATGGAGG**	**12**
40798	28	GGTATGGCAGGAAATTGATTGAACAAGG	8	40803	28	GGTATGGCAGGAAATTGATTGAACAAGG	8
40910	30	GGAGAAGGAAGGAGCAGAGGATTCAAAAGG	19	40915	30	GGAGAAGGAAGGAGCAGAGGATTCAAAAGG	19
40988	20	GGTGCATGGAGGAAGAAAGG	16	40993	20	GGTGCATGGAGGAAGAAAGG	16
41051	29	GGTGAGTGAAGGCAAGGCAAAATCAGTGG	14	41056	29	GGTGAGTGAAGGCAAGGCAAAATCAGTGG	14
41095	22	GGCCTGAAGGTAGAGGGGATGG	19	41100	22	GGCCTGAAGGTAGAGGGGATGG	19
41126	28	GGAAGGTAGTTCAGGAGCAGAACACTGG	12	41131	28	GGAAGGTAGTTCAGGAGCAGAACACTGG	12
41162	17	GGAGAGGGAGAAGGGGG	18	41167	17	GGAGAGGGAGAAGGGGG	18
41204	17	GGAGAGGCCAGGAGAGG	21	41209	17	GGAGAGGCCAGGAGAGG	21
**41251**	**27**	**GGAAGAAGAGGGATGGGGAAGAGCAGG**	**19**	**41248**	**26**	**GGAAGAATGGAAGAAGAGGGATGGGG**	**19**
**41285**	**26**	**GGAGAGGGAGCAGGGCCATCAGAAGG**	**16**	**41281**	**23**	**GGAGCAAAAGGAGAGGGAGCAGG**	**18**
**41330**	**14**	**GGAGGGAGGGGAGG**	**21**	**41332**	**20**	**GGAGGAGGGAGGGGAGGAGG**	**21**
**41345**	**26**	**GGAGCATGGAGAAGGAGAAGAAGAGG**	**16**	**41357**	**23**	**GGAGAAGGAGAAGAAGAGGAGGG**	**14**
41399	30	GGAGGGAGAAGGGAAAGAGGAAGGAGAAGG	19	41404	30	GGAGGGAGAAGGGAAAGAGGAAGGAGAAGG	19
41456	23	GGAGGAAGGAGAGAGGAAAAAGG	21	41461	23	GGAGGAAGGAGAGAGGAAAAAGG	21
**41491**	**21**	**GGAAGGAGGAGAAAGGAGAGG**	**19**	**41485**	**17**	**GGAAAGAGCGGGGAAGG**	**14**
41534	29	GGAAGAGGAGGAAACAGAGGGGAGAGGGG	21	41503	21	GGAGAAAGGAGAGGAAGAAGG	19
41576	23	GGGAGGGGAAGTAGAGGGAGGGG	37	41539	29	GGAAGAGGAGGAAACAGAGGGGAGAGGGG	21
**41609**	**23**	**GGGGAAAGGAGAGAGGGAAGAGG**	**21**	**41581**	**23**	**GGGAGGGGAAGTAGAGGGAGGGG**	**37**
**41636**	**23**	**GGAGGAGGGTGAGGGGGAAGAGG**	**21**	**41611**	**19**	**GGAGGGGAAAGGAGAGAGG**	**19**
				**41635**	**20**	**GGAAGAGGAGGAGGGTGAGG**	**21**
41664	30	GGGGAGGGGGAAGAGGAGGAAGGGGAGGGG	53	41669	30	GGGGAGGGGGAAGAGGAGGAAGGGGAGGGG	53
41699	23	GGAGGAAGGAGAAGGGAAAGGGG	21	41704	23	GGAGGAAGGAGAAGGGAAAGGGG	21
41723	26	GGAAGAAGGGGAAGAAGGAGAAGGGG	21	41728	26	GGAAGAAGGGGAAGAAGGAGAAGGGG	21
**41750**	**26**	**GGAAGAAGGGGAGGAAGGAGAAGGGG**	**21**	**41762**	**11**	**GGGGAGGAAGG**	**19**
41779	23	GGGAAGAGGAGGAAGGAGAAGGG	21	41777	23	GGGGAGGGGGAAGAGGAGGAAGG	21
**41805**	**21**	**GGAGAAGAGGAAGGAGAAGGG**	**17**	**41818**	**18**	**GGAAGGAGAAGGGGAGGG**	**19**
**41829**	**25**	**GGAGAAGAGGAGGAAGGAGAAGGGG**	**20**	**41842**	**21**	**GGAGGAAGGAGAAGGGGAGGG**	**20**
41864	18	GGAAGGAGAAGGGGAGGG	19	41869	18	GGAAGGAGAAGGGGAGGG	19
41888	23	GGAGGAAGGAGAAGGGAAAGGGG	21	41893	23	GGAGGAAGGAGAAGGGAAAGGGG	21
41915	15	GGAAGGAGAGGAAGG	20	41920	15	GGAAGGAGAGGAAGG	20
41934	23	GGGGAGGGGGAAGAGGAGGAAGG	21	41939	23	GGGGAGGGGGAAGAGGAGGAAGG	21
41961	30	GGGGAAGGGGAGGATGGAGAAGGGGAGGGG	53	41966	30	GGGGAAGGGGAGGATGGAGAAGGGGAGGGG	53
**41999**	**17**	**GGAAGGAGAATGGGAGG**	**18**	**42001**	**23**	**GGAGGAAGGAGAATGGGAGGGGG**	**21**
42016	23	GGGAAGAGGAGGAAGGAGAAGGG	21	42028	23	GGAGGAAGGAGAAGGGGAGGGGG	21
**42041**	**15**	**GGGGGAAGAGGAAGG**	**18**	**42065**			
42060	30	GGGGAAGGGGAGGAAGGAGAAGGGGAGGGG	53	42100	30	GGGGAAGGGGAGGAAGGAGAAGGGGAGGGG	53
42108	23	GGGGAGGGGGAAGAGGAGGAAGG	21	42127	23	GGAGGAAGGAGAAGGGGAGGGGG	21
**42131**	**24**	**GGAAGAAGAAGGGGAGGAAGAAGG**	**16**	**42146**	**11**	**GGAGGAAGGGG**	**19**
				**42163**	**14**	**GGGGAGGAAGAAGG**	**16**
42158	29	GGGAGAGGAAGAAGGGGAGGGAGAAGGGG	34	42193	29	GGGAGAGGAAGAAGGGGAGGGAGAAGGGG	34
**42197**	**20**	**GGAAGGGGAAGTGGAAGGGG**	**21**	**42214**	**17**	**GGAAGAAGAGGAAGGGG**	**14**
**42218**	**26**	**GGTGGAAGGGGAGGAAGGAGAGGGGG**	**21**	**42239**	**20**	**GGAAGGGGAGGTGGAAGGGG**	**21**
				**42266**	**19**	**GGAGAGGGGGAAGGAGAGG**	**20**
42261	14	GGAGAGGAGGAAGG	19	42295	14	GGAGAGGAGGAAGG	19
42290	12	GGAGGGGGAAGG	20	42318	12	GGAGGGGGAAGG	20
42313	21	GGAGGAACAGAGAAGAGGAGG	11	42340	21	GGAGGAACAGAGAAGAGGAGG	11
42335	30	GGAAGAAGAGGGGAAGTATCAGGAGACAGG	17	42387	30	GGAAGAAGAGGGGAAGTATCAGGAGACAGG	17
42382	15	GGCAGGATGGAGAGG	20	42774	15	GGCAGGATGGAGAGG	20
42769	21	GGAGGCACTGGTAAATTTTGG	14	44989	21	GGAGGCACTGGTAAATTTTGG	14
44984	20	GGGTTAGGGGTGGGTTGGGG	41	45620	20	GGGTTAGGGGTGGGTTGGGG	41
45615	26	GGAGGCAGATACCTGTATGGATCTGG	9	45805	26	GGAGGCAGATACCTGTATGGATCTGG	9
45800	26	GGAGGTGTGGGAGCATACTTACTAGG	9	45861	26	GGAGGTGTGGGAGCATACTTACTAGG	9
45856	22	GGTTTCAGAGAGGAAGGAGTGG	14	45907	22	GGTTTCAGAGAGGAAGGAGTGG	14
**45902**	**26**	**GGAGGCAGCCTAGTAGAGGGCCAGGG**	**10**	**46359**	**27**	**GGAGGCAGCCTAGTAGAGGGCCAGGGG**	**10**
				**46387**	**27**	**GGACACCTAGAAAAGTTGTGGTGGTGG**	**5**
46373	11	GGTGGTGGTGG	21	47096	11	GGTGGTGGTGG	21
47091	26	GGATTAGGAATGACATAAGGGAGTGG	15	47356	26	GGATTAGGAATGACATAAGGGAGTGG	15
47351	16	GGGAGGCTGAGGCTGG	19	47620	16	GGGAGGCTGAGGCTGG	19
47615	26	GGACTTTTGGAAAGCCTGGGTCTGGG	19	49610	26	GGACTTTTGGAAAGCCTGGGTCTGGG	19
49605	15	GGCTGGGCGTGGTGG	19	49650	15	GGCTGGGCGTGGTGG	19
49645	16	GGGAGGCCAAGGCGGG	19	49683	16	GGGAGGCCAAGGCGGG	19
49678	29	GGAGTTTGAGGCCAGCCTGGCCAACATGG	21	49750	29	GGAGTTTGAGGCCAGCCTGGCCAACATGG	21
**49745**	**13**	**GGTGTGGTGGTGG**	**19**	**49785**	**14**	**GGTGTGGTGGTGGG**	**19**
49780	16	GGGAGGCTGAGGCAGG	19	49818	16	GGGAGGCTGAGGCAGG	19
49813	11	GGAGGTGGAGG	21	51801	11	GGAGGTGGAGG	21
51796	24	GGAAGTAGAATGGTATGGGCCAGG	15	51896	24	GGAAGTAGAATGGTATGGGCCAGG	15
51891	28	GGAGCCCTGGCACTGGTGGCAGAAATGG	20	52635	28	GGAGCCCTGGCACTGGTGGCAGAAATGG	20
52630	29	GGAACATTTATGAATTCAGGTTAGGGTGG	7	55585	29	GGAACATTTATGAATTCAGGTTAGGGTGG	7
55580	27	GGCTGGAGTGCAATGGCGCGATCTTGG	14	55730	27	GGCTGGAGTGCAATGGCGCGATCTTGG	14
55725	25	GGTTTCTCCATGTTGGTCAGGCTGG	11	57350	25	GGTTTCTCCATGTTGGTCAGGCTGG	11
57345	21	GGAATCTCTGGGTTATCGGGG	14	57787	21	GGAATCTCTGGGTTATCGGGG	14
57782	29	GGATTACAGGCATGGGCTACCGCGCCTGG	14	57857	29	GGATTACAGGCATGGGCTACCGCGCCTGG	14
57852	12	GGAATGGGGAGG	18	59219	12	GGAATGGGGAGG	18
59213	27	GGCTGGAGTGCAGTGGCATAATCTCGG	14	59366	27	GGCTGGAGTGCAGTGGCATAATCTCGG	14
59360	25	GGTTTCACCATATTGGCCAGGCTGG	11	59513	25	GGTTTCACCATATTGGCCAGGCTGG	11

**Table 5:** G4 clusters found by G4P Calculator in wildtype control sample, treated and untreated proband's DNA.

Seqnames: X dna:chromosome chromosome:GRCh38:X:38268563:38328144: 1							WILDTYPE and TREATED		UNTREATED	
**width**	**strand**	**score**	**max_score**	**threshold**	**window**	**sequence (WILDTYPE and TREATED)**	**start**	**end**	**start**	**end**
37	–	−1.35	−1.7	1.5	20	GGCTAGAAAGGGTAGTGGGGTGTGTGTAGGAGGGAGG	12551	12587	12556	12592
27	+	1.78	2.2	1.5	20	GTTGGGGTTATGGGAACTGGGGGCAGG	13625	13651	13630	13656
25	+	1.52	1.95	1.5	20	GCTTCTAGGGGATTTAGGGGGCTGG	16326	16350	16331	16355
**19**	**+**	**1.58**	**1.5**	**1.5**	**20**	**GGGGAGGTTGAGGTTGAGG (UNTREATED)**	**/**	**/**	**18078**	**18096**
22	–	−1.77	−1.9	1.5	20	GTGACTTGGGGAGTGGGGGATG	26178	26199	26183	26204
18	+	1.72	1.5	1.5	20	GCTAGAGGGGGATGTGGG	34190	34207	34195	34212
29	+	1.21	1.5	1.5	20	GTGGATTTAAAGGGGAGGGATCAGTGTGG	35608	35636	35613	35641
**36**	**+**	**1.47**	**2.35**	**1.5**	**20**	**GAGTGTAGCTGGGGGTGGAGGGGGAGAGAGAGAGAG (WILDTYPE)** **GAGTGTAGCTGGGGGTGGTGGGGGAGAGAGAGAGAG (TREATED)**	**36135**	**36170**	**36140**	**36175**
32	+	1.44	1.8	1.5	20	GAGGAGAGGGAGAAGGGGGAGAAAGACAAGGG	41160	41191	41165	41196
29	+	1.28	1.55	1.5	20	GGAAGAAGAGGGATGGGGAAGAGCAGGAG	41251	41279	41256	41284
32	+	1.47	2.05	1.5	20	GAGATGGAGGAGGGAGGGGAGGAGGAGCATGG	41322	41353	41327	41358
22	+	1.5	1.6	1.5	20	GAAGGGGAAGAAGTGGAGGGAG	41424	41445	41429	41450
20	+	1.5	1.5	1.5	20	GGGGAAGGAGGAGAAAGGAG	41489	41508	41494	41513
103	+	1.61	2.15	1.5	20	GGAGAGGGGGAAGAGGAGGAAACAGAGGGGAGAGGGGAGGAAAAAGAGGAGGGAGGGGAAGTAGAGGGAGGGGAAGTAGAGGAGGGGAAAGGAGAGAGGGAAG	41526	41628	41531	41633
629	+	1.71	2.3	1.5	20	GGAAGAGGAGGAGGGTGAGGGGGAAGAGGAGGAAGGGGAGGGGGAAGAGGAGGAAGGGGAGGGGGAAGAGGAGGAAGGAGAAGGGAAAGGGGAGGAAGAAGGGGAAGAAGGAGAAGGGGAGGAAGAAGGGGAGGAAGGAGAAGGGGAGGGGGAAGAGGAGGAAGGAGAAGGGGAGGGAGAAGAGGAAGGAGAAGGGGAGGGAGAAGAGGAGGAAGGAGAAGGGGAGGGAGAAGAGGAAGGAGAAGGGGAGGGAGAAGAGGAGGAAGGAGAAGGGAAAGGGGAGGAGGAAGGAGAGGAAGGAGAAGGGGAGGGGGAAGAGGAGGAAGGAGAAGGGGAAGGGGAGGATGGAGAAGGGGAGGGGGAAGAGGAGGAAGGAGAATGGGAGGGGGAAGAGGAGGAAGGAGAAGGGGAGGGGGAAGAGGAAGGAGAAGGGGAAGGGGAGGAAGGAGAAGGGGAGGGGGAAGAGGAGGAAGGAGAAGGGGAGGGGGAAGAGGAGGAAGGGGAAGAAGAAGGGGAGGAAGAAGGAGAGGGAGAGGAAGAAGGGGAGGGAGAAGGGGAGGAAGAAGAGGAAGGGGAAGTGGAAGGGGAGGTGGAAGGGGAGGAAGGAGAGGGGGAAGGAGAGGAAGAGG	41630	42258	41635	42263
28	+	1.43	1.85	1.5	20	GAAAGGGAAAAGGAGGGGGAAGGAGAAG	42279	42306	42284	42311
19	+	1.58	1.5	1.5	20	GGAGGAGGAAGAAGAGGGG	42329	42347	42334	42352
35	+	1.49	2.5	1.5	20	GTATTGTGGGTTAGGGGTGGGTTGGGGTACAAAAG	44977	45011	44982	45016
24	–	−1.62	−2.1	1.5	20	GGGAACTGGGGAGAGGGGTGCCAG	47683	47706	47688	47711
20	–	−1.75	−1.75	1.5	20	GCAGTATGGGGTGGGAAGGG	48146	48165	48151	48170
44	–	−1.91	−2.95	1.5	20	GGAGAATGCTGGGATGGGGGCTGGGGGGTGGGGCTGAGAGTGGG	48577	48620	48582	48625
16	+	1.88	1.5	1.5	20	GAAAAGTATGGGGGGG	50582	50597	50587	50602
29	–	−1.62	−2.05	1.5	20	GTTAGGGAAAAAGGGGAAGGGGAATGGAG	52382	52410	52387	52415
20	–	−1.6	−1.6	1.5	20	GGGGGTAAATAACTGGGTGG	53782	53801	53787	53806
27	–	−1.26	−1.75	1.5	20	GCTGCTCAGGGGAGGCTGGGGCTGCAG	57405	57431	57410	57436
28	–	−1.39	−1.65	1.5	20	GGAAAGGAGTAGGGGCTGGGCACGGTGG	59452	59479	59458	59485

**Table 6:** G4 clusters found by G4 catchall in wildtype control sample, treated and untreated proband's DNA.

Sequence Definition: X dna:chromosome chromosome:GRCh38:X:38268563:38328144: 1					WILDTYPE and TREATED		UNTREATED	
**Given sequence (+ strand)**	**G4-forming sequence**	**Length**	**Strand**	**G4HScore**	**Beginning of G4 sequence**	**End of G4 sequence**	**Beginning of G4 sequence**	**End of G4 sequence**
GAGTTGGGGTTATGGGAACTGGGGGCAGG	GAGTTGGGGTTATGGGAACTGGGGGCAGG	29	+	1.69	13622	13651	13627	13656
GGGAGGGGAAGTAGAGGGAGGGGAAGTAGAGGAGGGG	GGGAGGGGAAGTAGAGGGAGGGGAAGTAGAGGAGGGG	37	+	2	41575	41612	41580	41617
GGGTGAGGGGGAAGAGGAGGAAGGGGAGGGGGAAGAGGAGGAAGGGGAGGGGG	GGGTGAGGGGGAAGAGGAGGAAGGGGAGGGGGAAGAGGAGGAAGGGGAGGGGG	53	+	2.264	41641	41694	41646	41699
GGGAAAGGGGAGGAAGAAGGGG	GGGAAAGGGGAGGAAGAAGGGG	22	+	2.091	41711	41733	41716	41738
GGGGAGGAAGGAGAAGGGGAGGGGG	GGGGAGGAAGGAGAAGGGGAGGGGG	25	+	2.44	41756	41781	41761	41786
GGGGAAGGGGAGGATGGAGAAGGGGAGGGGG	GGGGAAGGGGAGGATGGAGAAGGGGAGGGGG	31	+	2.484	41960	41991	41965	41996
GGGGAAGGGGAGGAAGGAGAAGGGGAGGGGG	GGGGAAGGGGAGGAAGGAGAAGGGGAGGGGG	31	+	2.484	42059	42090	42064	42095
GGGGAGGGGGAAGAGGAGGAAGGGG	GGGGAGGGGGAAGAGGAGGAAGGGG	25	+	2.44	42107	42132	42112	42137
GGGAGAGGAAGAAGGGGAGGGAGAAGGGGAGG	GGGAGAGGAAGAAGGGGAGGGAGAAGGGGAGG	32	+	1.906	42157	42189	42162	42194
GTGTATTGTGGGTTAGGGGTGGGTTGGGG	GTGTATTGTGGGTTAGGGGTGGGTTGGGG	29	+	1.828	44974	45003	44979	45008
CCCTCCTACACACACCCCACTACCC	GGGTAGTGGGGTGTGTGTAGGAGGG	25	–	−1.68	12553	12578	12558	12583
CCCCTCTCCCCAGTTCCCATGGCTCCTTCCC	GGGAAGGAGCCATGGGAACTGGGGAGAGGGG	31	–	−1.645	47688	47719	47693	47724
CCCACTCTCAGCCCCACCCCCCAGCCCCCATCCCAGCATTCTCC	GGAGAATGCTGGGATGGGGGCTGGGGGGTGGGGCTGAGAGTGGG	44	–	−1.909	48576	48620	48581	48625
CTCCATTCCCCTTCCCCTTTTTCCC	GGGAAAAAGGGGAAGGGGAATGGAG	25	–	−1.84	52381	52406	52386	52411

**Table 7:** G4 clusters found by ImGQfinder in wildtype control sample, treated and untreated proband's DNA.

*# Number of tetrads = 4*		WILDTYPE and TREATED				UNTREATED			
SEQ	defective G-run	G-run1 start	G-run2 start	G-run3 start	G-run4 start	G-run1 start	G-run2 start	G-run3 start	G-run4 start
GGGG GAAGAG GAGG AAACAGA GGGG AGA GGGG	2	41531	41541	41552	41559	41536	41546	41557	41564
GGGG AAGAG GAGG AAACAGA GGGG AGA GGGG	2	41532	41541	41552	41559	41537	41546	41557	41564
GGGG GAA GAGG AGGAA GGGG A GGGG	2	41648	41655	41664	41669	41653	41660	41669	41674
GGGG GAA GAGG AGGAA GGGG AG GGGG	2	41648	41655	41664	41670	41653	41660	41669	41675
GGGG GAAGA GGAG GAA GGGG A GGGG	2	41648	41657	41664	41669	41653	41662	41669	41674
GGGG GAAGA GGAG GAA GGGG AG GGGG	2	41648	41657	41664	41670	41653	41662	41669	41675
GGGG GAAGAG GAGG AA GGGG A GGGG	2	41648	41658	41664	41669	41653	41663	41669	41674
GGGG GAAGAG GAGG AA GGGG AG GGGG	2	41648	41658	41664	41670	41653	41663	41669	41675
GGGG AA GAGG AGGAA GGGG A GGGG	2	41649	41655	41664	41669	41654	41660	41669	41674
GGGG AA GAGG AGGAA GGGG AG GGGG	2	41649	41655	41664	41670	41654	41660	41669	41675
GGGG AAGA GGAG GAA GGGG A GGGG	2	41649	41657	41664	41669	41654	41662	41669	41674
GGGG AAGA GGAG GAA GGGG AG GGGG	2	41649	41657	41664	41670	41654	41662	41669	41675
GGGG AAGAG GAGG AA GGGG A GGGG	2	41649	41658	41664	41669	41654	41663	41669	41674
GGGG AAGAG GAGG AA GGGG AG GGGG	2	41649	41658	41664	41670	41654	41663	41669	41675
GGGG A GGGG GAA GAGG AGGAA GGGG	3	41664	41669	41676	41685	41669	41674	41681	41690
GGGG A GGGG GAAGA GGAG GAA GGGG	3	41664	41669	41678	41685	41669	41674	41683	41690
GGGG A GGGG GAAGAG GAGG AA GGGG	3	41664	41669	41679	41685	41669	41674	41684	41690
GGGG A GGGG GAAGAG GAGG AAGGGGA GGGG	3	41664	41669	41679	41690	41669	41674	41684	41695
GGGG AG GGGG AA GAGG AGGAA GGGG	3	41664	41670	41676	41685	41669	41675	41681	41690
GGGG AG GGGG AAGA GGAG GAA GGGG	3	41664	41670	41678	41685	41669	41675	41683	41690
GGGG AG GGGG AAGAG GAGG AA GGGG	3	41664	41670	41679	41685	41669	41675	41684	41690
GGGG AG GGGG AAGAG GAGG AAGGGGA GGGG	3	41664	41670	41679	41690	41669	41675	41684	41695
GGGG GAA GAGG AGGAA GGGG A GGGG	2	41669	41676	41685	41690	41674	41681	41690	41695
GGGG GAA GAGG AGGAA GGGG AG GGGG	2	41669	41676	41685	41691	41674	41681	41690	41696
GGGG GAAGA GGAG GAA GGGG A GGGG	2	41669	41678	41685	41690	41674	41683	41690	41695
GGGG GAAGA GGAG GAA GGGG AG GGGG	2	41669	41678	41685	41691	41674	41683	41690	41696
GGGG GAAGAG GAGG AA GGGG A GGGG	2	41669	41679	41685	41690	41674	41684	41690	41695
GGGG GAAGAG GAGG AA GGGG AG GGGG	2	41669	41679	41685	41691	41674	41684	41690	41696
GGGG AA GAGG AGGAA GGGG A GGGG	2	41670	41676	41685	41690	41675	41681	41690	41695
GGGG AA GAGG AGGAA GGGG AG GGGG	2	41670	41676	41685	41691	41675	41681	41690	41696
GGGG AAGA GGAG GAA GGGG A GGGG	2	41670	41678	41685	41690	41675	41683	41690	41695
GGGG AAGA GGAG GAA GGGG AG GGGG	2	41670	41678	41685	41691	41675	41683	41690	41696
GGGG AAGAG GAGG AA GGGG A GGGG	2	41670	41679	41685	41690	41675	41684	41690	41695
GGGG AAGAG GAGG AA GGGG AG GGGG	2	41670	41679	41685	41691	41675	41684	41690	41696
GGGG AGGAA GGAG AA GGGG A GGGG	2	41757	41766	41772	41777	41762	41771	41777	41782
GGGG AGGAA GGAG AA GGGG AG GGGG	2	41757	41766	41772	41778	41762	41771	41777	41783
GGGG AA GGGG AGGAT GGAG AA GGGG	3	41961	41967	41976	41982	41966	41972	41981	41987
GGGG AA GGGG AGGAT GGAG AAGGGGA GGGG	3	41961	41967	41976	41987	41966	41972	41981	41992
GGGG AGGAT GGAG AA GGGG A GGGG	2	41967	41976	41982	41987	41972	41981	41987	41992
GGGG AGGAT GGAG AA GGGG AG GGGG	2	41967	41976	41982	41988	41972	41981	41987	41993
GGGG AA GGGG AGGAA GGAG AA GGGG	3	42060	42066	42075	42081	42065	42071	42080	42086
GGGG AA GGGG AGGAA GGAG AAGGGGA GGGG	3	42060	42066	42075	42086	42065	42071	42080	42091
GGGG AGGAA GGAG AA GGGG A GGGG	2	42066	42075	42081	42086	42071	42080	42086	42091
GGGG AGGAA GGAG AA GGGG AG GGGG	2	42066	42075	42081	42087	42071	42080	42086	42092
GGGG A GGGG GAA GAGG AGGAA GGGG	3	42108	42113	42120	42129	42113	42118	42125	42134
GGGG A GGGG GAAGA GGAG GAA GGGG	3	42108	42113	42122	42129	42113	42118	42127	42134
GGGG A GGGG GAAGAG GAGG AA GGGG	3	42108	42113	42123	42129	42113	42118	42128	42134
GGGG AG GGGG AA GAGG AGGAA GGGG	3	42108	42114	42120	42129	42113	42119	42125	42134
GGGG AG GGGG AAGA GGAG GAA GGGG	3	42108	42114	42122	42129	42113	42119	42127	42134
GGGG AG GGGG AAGAG GAGG AA GGGG	3	42108	42114	42123	42129	42113	42119	42128	42134

A Spearman's rank correlation was utilized to assess the relationship between the detected variants and the location of predicted PQS, given the non-parametric nature of the genomic data. Our analysis revealed a moderate correlation (Spearman's rho = 0.55, p < 0.05), suggesting a potential link between the presence of variants and the formation or stabilization of PQS within the *RPGR* gene. This correlation was visually supported by a genomic map overlaying the detected variants onto the locations of predicted PQS, highlighting regions of overlap and potential functional significance.

### 3.7 The in-silico prediction analyses corroborated the hypothesis that G4 clusters determined by RPGR variants may alter the accessibility of the gene by DNA- and RNA-polymerases

**Fig. 5:**
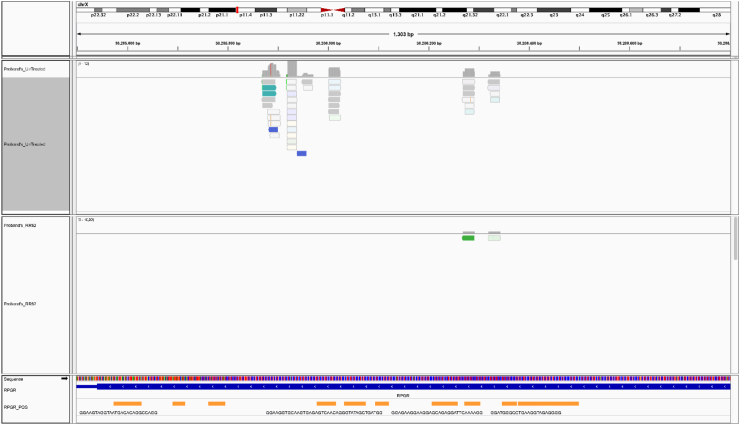
**Visualization of PQS within the *ORF15* Region of the *RPGR* Gene.** This figure illustrates the Integrative Genomics Viewer (IGV) representation of the *ORF15* region in the *RPGR* gene on the X chromosome, highlighting the distribution of predicted G-quadruplex structures (PQS). The tracks from top to bottom display the depth from sequencing experiments of both untreated and RR82-treated proband's samples, followed by the genomic sequence of *ORF15* and by a custom track representing PQS from all exploited computational tools. Each predicted structure is annotated with its respective position, providing insights into the potential formation sites of G-quadruplexes within this critical region associated with X-linked retinal degenerations. Due to graphical limitations, the sequences of PQS are not fully shown in the figure. This outcome likely illustrates the effects of pyridostatin treatment, leading to the diminished amplification of G4-forming regions and, consequently, the associated sequence data. Such results underscore the complexity and density of G-quadruplex forming sequences, suggesting their possible regulatory roles in gene expression and potential impact on the genetic pathogenesis of retinal disorders.

**Fig. 6:**
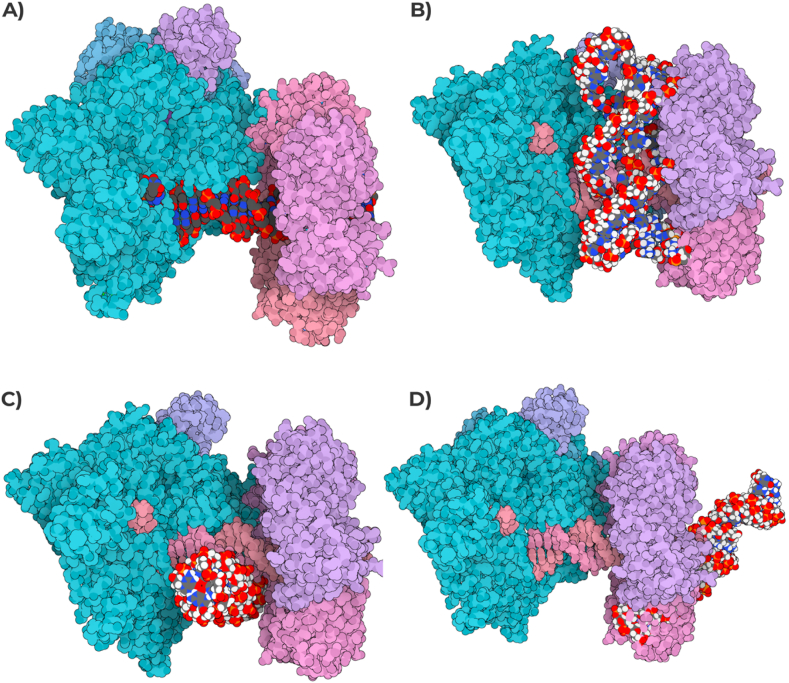
**The influence of putative G-Quadruplex structures on *RPGR* gene by 3D dynamic modeling.** G-quadruplex formed on *RPGR* in pyridostatin-treated samples may obstruct DNA polymerase activity, causing incorrect DNA tract placement and hindered DNA synthesis, with a noticeable impact in patient samples. A) Wildtype untreated sample. B) Wildtype pyridostatin treated sample. C) Proband's DNA pyridostatin treated sample. D) Proband's DNA untreated sample.

**Fig. 7:**
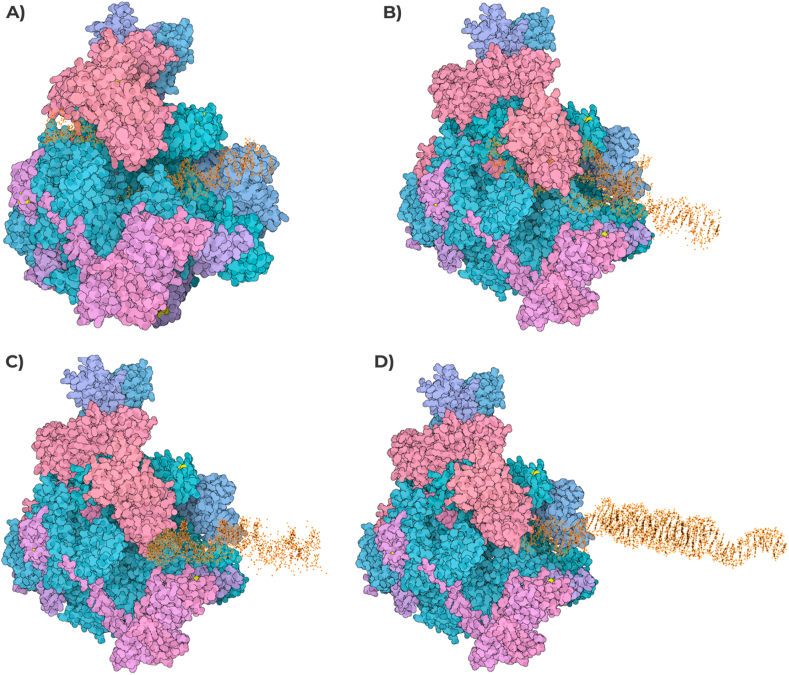
**The predicted impact of G-Quadruplex structures on *RPGR* gene transcription**. G-quadruplexes in treated samples may stall *RPGR* gene transcription at early stages. Notably, patient's treated samples may directly interfere with initial RNA polymerase binding, potentially causing altered gene expression levels or alternative splicing events with considerable phenotypic implications. A) Wildtype untreated sample. B) Proband's DNA untreated sample C) Wildtype pyridostatin treated sample. D) Proband's DNA pyridostatin treated sample.

The combined approach we used to predict DNA's potential PQS within *RPGR* sequence, in wildtype, pyridostatin treated and untreated samples globally highlighted huge differences in start/end of G4 clusters, as well as several sequence changes and number of PQS (Fig. 5). These changes in the start/end positions of PQS in pyridostatin-treated DNA might be attributable to the inhibition of polymerase extension across G4 motifs by pyridostatin. In untreated samples, polymerases can bypass these motifs more readily, leading to a different representation of PQS in sequencing data. The treatment with pyridostatin selectively stabilizes G4 structures, altering their detection and the resulting sequence data. The pqsfinder algorithm found 37 PQS in all samples, of which 25 with start/end in different positions between wildtype/treated and untreated samples, and one G4 forming sequence different between the same samples ([GGG(AGTGCAC)G]AGTGTAGCT[GGGG][GtGG]A[GGGG] in Wildtype/Treated and [GGG(AGTGCAC)G]AGTGTAGCT[GGGG][GtGG]T[GGGG] in Untreated) (Table 3). QGRS Mapper identified 177 PQS in wildtype/treated samples and 183 PQS in untreated one. Seven sequences of untreated found PQS resulted absent in wildtype/treated samples, while 144 PQS highlighted different start/end positions between the same samples (Table 4). The G4P Calculator found 104 G-runs in wildtype/treated samples and 103 in untreated one, without any block of unknown sequences. G4 Hunter detected 37 PQS in wildtype/treated samples and 38 in untreated one. 36 of the wildtype/treated PQS showed the same sequence of untreated counterparts but with different start/end positions, while one sequence is totally different between the considered samples (Table 5). The G4 catchall identified 24 PQS in all samples, with 10 showing the same sequence but with different start/end positions, and four also presenting different sequence between wildtype/treated and untreated samples (Table 6). Furthermore, ImGQfinder detected 50 PQS in all samples, with the same sequence but with different start/end positions between wildtype/treated and untreated samples (Table 7). What was found by G4 sequence prediction tools was very interestingly confirmed by 3D interacting simulations from 3D Nus and SAMSON Connect. G-quadruplex structures have been shown to cause stalling of the replication fork, an effect attributed to the physical impediment posed by these structures. The DNA polymerase may encounter difficulty in “unwinding” these structures, which could potentially lead to incomplete or erroneous replication, creating genetic instability. Found data on the *RPGR* gene has indicated an abundance of potential G-quadruplex-forming sequences. As evidenced in Fig. 6, the putative G-Quadruplex formed on *RPGR* of samples treated with pyridostatin might alter the activity of DNA Polymerase, putting the DNA tract in an incorrect way and blocking the DNA synthesis, especially on patient's one. Therefore, it is plausible that these structures could influence the binding and processivity of DNA polymerase during replication. Additionally, the RNA polymerase, responsible for transcribing DNA into RNA, may also be influenced by the presence of G-quadruplex structures. G-quadruplexes situated within gene promoter regions could interfere with the binding and initiation of RNA polymerase, consequently impacting gene expression. For genes such as *RPGR*, where precise expression levels are critical, the formation of G-quadruplexes may significantly influence their regulation. Already described *in vitro* studies with synthetic templates have shown that the presence of a G-quadruplex can cause pausing or termination of transcription by RNA polymerase. This effect depends on the stability and location of the G-quadruplex and the specific RNA polymerase involved. As highlighted in Fig. 7, the treated samples might arrest the *RPGR* gene transcription after the first nucleotides and, more interestingly, the patient's treated one might directly interfere with RNA polymerase initial binding. This could lead to altered expression levels or alternative splicing events, with potentially significant phenotypic consequences.

## 4 Discussion

Our focus was on a Sicilian family, who exhibited typical symptoms of IRD and showed an X-linked transmission pattern. However, we did not find any causative mutations in the two known genes, *RP2* and *RPGR*, nor in other potentially implicated candidate genes located on the X chromosome. Moreover, upon analyzing other known IRD causative genes listed in RetNet database (https://web.sph.uth.edu/RetNet/), none were found to carry noteworthy variants. This led us to hypothesize the possibility of an epigenetic mechanism influencing the condition. Since the *RPGR* is known to present several regions that form G4 structures and harbored multiple variants (even if not directly involved, individually, in the RPGR protein functionality) in the proband, we decided to concentrate our investigation on this gene. Our findings provide novel insights into the formation of G4-quadruplex structures within the *RPGR* gene and their potential impacts on DNA replication and transcription in the molecular pathogenesis of RPGR-related retinal degeneration. We demonstrated that the G4-stabilizing ligand pyridostatin selectively inhibits DNA polymerase extension on known G4-forming sequences during PCR amplification. This finding aligns with previous studies showing stalled polymerase progression at stabilized G4 structures [32]. The observed correlation between genomic variants and PQS within the RPGR gene suggests a nuanced relationship between genetic variations and the propensity for G-quadruplex formation, potentially influencing the gene's transcriptional and replication processes. This relationship is particularly significant given the role of the RPGR gene in retinal function and its association with X-linked Retinitis Pigmentosa. G4 motifs can form highly stable secondary structures involving planar arrays of four guanine bases stabilized by Hoogsteen hydrogen bonding. The unusual topology of these non-canonical DNA conformations is thought to impede polymerase binding and translocation [33]. Our work confirms the ability of stabilized G4 quadruplexes to obstruct DNA synthesis by polymerase enzymes. Subsequent whole genome amplification experiments further supported widespread G4-mediated blockade of polymerase activity across the human genome. A marked reduction in WGA product yield was observed when amplification was performed in the presence of the G4-stabilizing pyridostatin compound. Computational algorithms predict over 700,000 potential G4-forming sequences scattered throughout the human genome [34]. Several recent studies using next-generation sequencing approaches similarly demonstrated reduced amplification of genomic regions neighboring predicted G4 motifs stabilized by G4-binding ligands [16]. Our WGA data reinforces that DNA polymerases likely encounter consistent impedance at the multitude of genomic G4 structures stabilized by pyridostatin. Through high-throughput sequencing of WGA products, we identified clusters of enriched or depleted read counts coinciding with putative G4-forming regions. Regions of reduced amplification, such as the known G4-forming sequences in *c-MYC* and *c-KIT*, correspond to sites of polymerase blockage at stabilized G4 motifs. Most strikingly, sequencing of the *RPGR* gene showed a marked reduction from 24 variants detected in untreated DNA to just 7 variants in pyridostatin-treated DNA. Interestingly, the presence of intronic variants in regions predicted to form G-quadruplex structures may indicate a mechanism by which genetic instability or altered gene expression contributes to disease pathology. The reduction in detected variants upon pyridostatin treatment further emphasizes the role of G4 structures in mediating these effects. Our findings align with emerging literature on the role of G-quadruplexes in genetic regulation and disease, suggesting that the stabilization of these structures could have both protective and deleterious effects depending on the genomic context [35]. RPGR plays an essential role in retinal photoreceptor function, and mutations are associated with more than 70 % of X-linked retinal degeneration cases [36]. Moreover, recent studies have identified several important *cis*-regulatory elements and transcription factors involved in controlling *RPGR* transcription, whose alteration could also lead to IRDs: 1) the *RPGR* promoter lacks a TATA box but contains a conserved CpG island characteristic of housekeeping genes. Binding sites for general transcription factors like Sp1 have been identified; 2) photoreceptor-specific expression is mediated through multiple redundant enhancer elements spread throughout the gene's introns. These enhancers are bound by photoreceptor transcription factors like CRX and NRL; 3) additional retinal transcription factors like NR2E3 can modulate *RPGR* expression by competing for overlapping enhancer binding sites; 4) the intron 15 G4 motif cluster may also contribute unique regulatory features, as G4 structures are known to influence transcription factor binding; 5) Epigenetic changes including DNA methylation of the promoter CpG island provide another layer of *RPGR* regulation and are disrupted in some retinal dystrophies; 6) miRNAs like miR-3619-5p have been found to directly target and downregulate *RPGR* [37]. This complex multi-level regulation allows precise spatiotemporal control of *RPGR* expression, which is vital for photoreceptor function. Our data implies that stabilized G4 structures extensively throughout *RPGR* likely obstructed the DNA polymerase, preventing complete replication and variant detection. As already cited, the *RPGR* gene presents one of the highest densities of predicted G4-forming motifs in the human genome, concentrated particularly within intron 15 [38]. Computational prediction tools corroborated a high propensity for G4 formation throughout *RPGR*, with pyridostatin treatment shifting the start/end sites and sequences of many putative G4 motifs. 3D molecular modeling vividly demonstrated the potential for these dynamic G4 structures to introduce abnormalities in DNA topology that could interfere with polymerase binding and progression along the gene. Replication fork stalling and collapse at G4 roadblocks can lead to DNA double-strand breaks, deletion mutations, and chromosome rearrangements through erroneous DNA repair [39]. One proposed disease mechanism in triplet repeat expansion disorders like Huntington's disease implicates DNA secondary structures including G4 motifs promoting slippage of the replication fork and trinucleotide repeat expansions [40]. Our integrated data indicates that aberrant *RPGR* replication due to pervasive G4 formation may similarly stimulate genetic instability. G4 structures within promoter regions could also profoundly impact transcription. G4 motifs have been shown to influence gene expression by altering transcription factor binding sites or interfering with assembly of the preinitiation complex [41]. In many genes, G4 presence causes premature transcription termination, though some genes exhibit enhanced expression from stabilized G4 structures [42]. Computational and 3D modeling indicated potential for G4 formation to disrupt critical RPGR promoter elements and RNA polymerase binding, drastically reducing gene expression. Disrupted transcriptional regulation of *RPGR*, which is finely tuned in photoreceptors [43], likely contributes to the retinal dystrophy phenotype. Overall, our integrated approach revealed a widespread distribution of stabilized G4-quadruplex structures throughout the *RPGR* gene that could profoundly disrupt DNA and RNA polymerase processes. Moreover, the correlation between detected changes and PQS underscores the potential for G-quadruplex-targeted therapies. By modulating the formation or stabilization of these structures, it may be possible to influence gene expression patterns and mitigate disease progression.

## 5 Conclusions

Aberrant replication and transcription resulting from pervasive G4 formation may represent a key molecular pathological mechanism underlying vision loss in *RPGR*-related retinal degeneration. Elucidating the dynamics of *RPGR* G4 motifs may reveal novel targets for therapies aimed at normalizing gene transcription through stabilization or resolution of pathogenic G4 structures. However, our current understanding of these complex structures and their mechanisms of action was mainly supported by *in silico* modelling analyses, and necessitates further comprehensive research, and the precise effects of G4 structures on *RPGR* gene regulation and function warrants further *in vitro* and *in vivo* functional investigation [44,45]. While our study is *RPGR*-centric, it is crucial to acknowledge that the implications of G-quadruplexes in genetic pathologies extend beyond this gene. Thus, the understanding gleaned from this research can serve as a foundation for future explorations into the role of G-quadruplexes in other genetic disorders [35]. In conclusion, our study substantially contributes to the burgeoning body of evidence supporting the role of G-quadruplex structures in genetic pathologies, offering a unique perspective on their potential involvement in *RPGR*-associated disorders. We anticipate that as the scientific understanding of G-quadruplex structures continues to evolve, so too will the development of innovative therapeutic strategies to combat genetic disorders, marking a significant stride towards personalized medicine and precision health.

## Ethics statement

This study was approved by the ethics committee of the “Azienda Policlinico Universitario di Messina” (record number: 23/17bis). All procedures involving human participants were performed in accordance with institutional and/or national research committee ethical standards and performed in accordance with the 1964 Declaration of Helsinki and its later amendments or comparable ethical standards. The participants gave written informed consent for publication of their clinical exams.

## Data availability statement

Data will be made available on request.

## Funding

This research did not receive any specific grant from funding agencies in the public, commercial, or not-for-profit sectors.

## CRediT authorship contribution statement

**Luigi Donato:** Writing – original draft, Methodology, Formal analysis, Data curation, Conceptualization. **Concetta Scimone:** Conceptualization. **Simona Alibrandi:** Conceptualization. **Domenico Mordà:** Conceptualization. **Ivan Anchesi:** Conceptualization. **Sergio Zaccaria Scalinci:** Formal analysis, Data curation, Conceptualization. **Carmela Rinaldi:** Conceptualization. **Rosalia D'Angelo:** Writing – review & editing, Supervision. **Antonina Sidoti:** Writing – review & editing, Supervision.

## Declaration of competing interest

The authors declare that they have no known competing financial interests or personal relationships that could have appeared to influence the work reported in this paper.
